# Supplementary material for: No relationship between fornix and cingulum degradation and within-network decreases in functional connectivity in prodromal Alzheimer’s disease
Source: PLoS One. 2019 Oct 3;14(10):e0222977. doi: 10.1371/journal.pone.0222977 (PMC6776361; doi:10.1371/journal.pone.0222977)
Supplement: S1 File — (DOCX) [file pone.0222977.s001.docx]

**Supplemental Information to:**

No relationship between fornix and cingulum degradation and within-network decreases in functional connectivity in preclinical Alzheimer’s disease

Gilligan, T.M., Sibilia, F., Farrell, D., Lyons, D., Kennelly, S.P., Bokde, A. L. W.

**Neuropsychological Data**

**S1 Table A. CERAD tests normed for age, education and gender.** (Mean + SD. P-value: 1-sided, uncorrected).

| Measure | HC | MCI | Statistic (Y_t_) | p-value |
| --- | --- | --- | --- | --- |
| MMSE | 0.270 ± 0.652 | -1.72 ± 2.16 | -3.47 | .003 |
| Fluency | 0.067 ± 0.824 | -0.351 ± 1.23 | -1.62 | .058 |
| Naming | 0.211 ± 0.619 | -2.09 ± 3.21 | -3.06 | .012 |
| Word Delay | 0.997 ± 0.735 | -1.60 ± 1.49 | -5.96 | .000 |
| Word Recognition Y | 0.530 ± 0.218 | -1.37 ± 2.29 | -3.08 | .016 |
| Word Recognition N | 0.339 ± 0.078 | -0.766 ± 2.19 | -1.43 | .068 |
| Praxis | 0.478 ± 0.527 | 0.212 ± 0.624 | -1.62 | .043 |
| Trail A | 0.266 ± 0.679 | -1.08 ± 1.46 | -3.40 | .003 |
| Trail B | 0.491 ± 0.847 | -2.28 ± 2.37 | -5.40 | .000 |


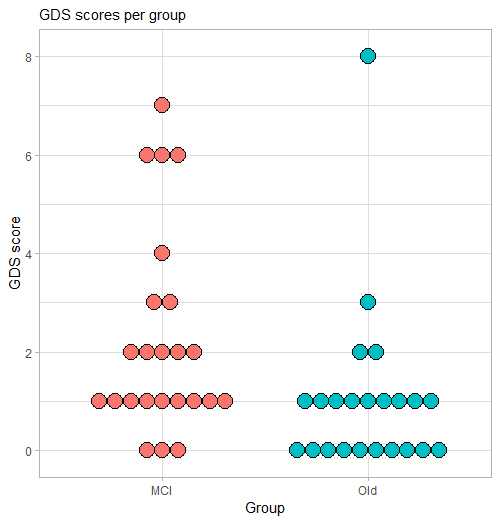


**S1 Fig A. Depression scores were significantly higher in the MCI compared to the HC group.**


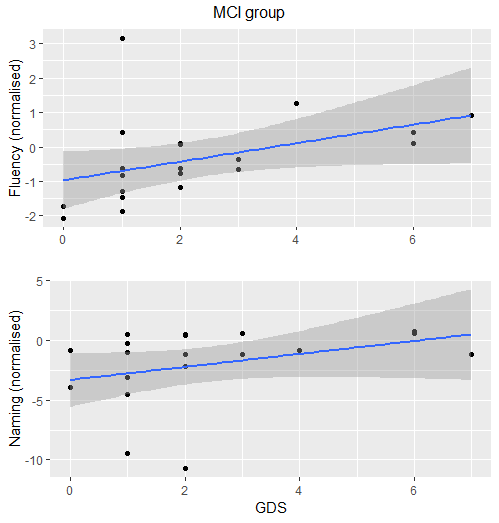


**S1 Fig B. Both naming and fluency scores were correlated with depression in the MCI group, plotting shows those relationships to be positive.**

**DWI Measures**

**S1 Table B. Fornix** (P-value: uncorrected, 1-sided)

| Fornix | Measure | HC | MCI | Est | *t*-statistic | p-value |
| --- | --- | --- | --- | --- | --- | --- |
| Left | FA | 0.282 ± 0.032 | 0.262 ± 0.039 | -0.028805 | -2.784 | 0.003975 |
|  | MD | 0.00120 ± 0.00008 | 0.00132 ± 0.00014 | 0.000139 | 4.210 | 0.000064 |
|  | Da | 0.00159 ± 0.00007 | 0.00171 ± 0.00012 | 0.0001297 | 5.32 | 0.000002 |
|  | Dr | 0.00101 ± 0.00009 | 0.00112 ± 0.00015 | 0.0001435 | 3.74 | 0.000269 |
|  | Volume | .0016 ± 0.00048 | 0.0016 ± 0.00066 | -0.000103 | -0.489 | 0.313660 |
| Right | FA | 0.281 ± 0.032 | 0.273 ± 0.031 | -0.016815 | -2.419 | .009925 |
|  | MD | 0.00118 ± 0.00008 | 0.00127 ± 0.00008 | 0.000100 | 5.056 | .00000422 |
|  | Da | 0.00156 ± 0.00006 | 0.00167 ± 0.00007 | 0.0001079 | 5.294 | .0000019 |
|  | Dr | 0.00100 ± 0.00009 | 0.00107 ± 0.00009 | 0.0000977 | 4.653 | .0000156 |
|  | Volume | 0.0019 ± 0.00058 | 0.0018 ± 0.0007 | -0.000219 | -1.225 | .1137 |

**S1 Table C. Parahippocampal Cingulum** (P-value: uncorrected, 1-sided)

| Para-hippocampal  Cingulum | Measure | HC | MCI | Est | *t*-statistic | p-value |
| --- | --- | --- | --- | --- | --- | --- |
| Left | FA | 0.344 ± 0.025 | 0.338 ± 0.019 | -0.002792 | -0.399 | 0.34585 |
|  | MD | 0.00072 ± 0.00003 | 0.00075 ± 0.00005 | 0.0000187 | 2.721 | 0.00467 |
|  | Da | 0.00099 ± 0.00003 | 0.00103 ± 0.00005 | 0.0000238 | 3.016 | 0.00215 |
|  | Dr | 0.00058 ± 0.00003 | 0.00061 ± 0.00005 | 0.0000181 | 2.194 | 0.01685 |
|  | Volume | 0.001 ± 0.0004 | 0.001 ± 0.0004 | 0.0000323 | 0.288 | 0.38750 |
| Right | FA | 0.361 ± 0.029 | 0.355 ± 0.022 | -0.005139 | -0.728 | 0.23535 |
|  | MD | 0.00072 ± 0.00003 | 0.00074 ± 0.00005 | 0.0000104 | 1.007 | 0.15950 |
|  | Da | 0.00101 ± 0.00003 | 0.00103 ± 0.00006 | 0.00000942 | 0.865 | 0.19600 |
|  | Dr | 0.00057 ± 0.00003 | 0.00059 ± 0.00005 | 0.00001318 | 1.176 | 0.12300 |
|  | Volume | 0.0014 ± 0.0005 | 0.0011 ± 0.0003 | -0.0002405 | -1.860 | 0.03485 |

**S1 Table D. Retrosplenial cingulum** (P-value: uncorrected, 1-sided)

| Retrosplenial Cingulum | Measure | HC | MCI | Est | *t*-statistic | p-value |
| --- | --- | --- | --- | --- | --- | --- |
| Left | FA | 0.429 ± 0.021 | 0.425 ± 0.037 | -0.006141 | -0.675 | 0.25150 |
|  | MD | 0.00067 ± 0.00002 | 0.00069 ± 0.00002 | 0.0000213 | 3.084 | 0.00178 |
|  | Da | 0.00100 ± 0.00003 | 0.00103 ± 0.00004 | 0.0000224 | 1.576 | 0.06100 |
|  | Dr | 0.00050 ± 0.00002 | 0.00052 ± 0.00003 | 0.0000199 | 2.432 | 0.00965 |
|  | Volume | 0.002 ± 0.0005 | 0.002 ± 0.0008 | 0.0000751 | 0.338 | 0.36850 |
| Right | FA | 0.406 ± 0.019 | 0.406 ± 0.040 | -0.009380 | -1.232 | 0.11250 |
|  | MD | 0.00067 ± 0.00002 | 0.00068 ± 0.00003 | 0.0000148 | 2.052 | 0.02315 |
|  | Da | 0.00098 ± 0.00003 | 0.00099 ± 0.00003 | 0.0000184 | 1.881 | 0.03339 |
|  | Dr | 0.00051 ± 0.00002 | 0.00053 ± 0.00004 | 0.0000177 | 1.692 | 0.04895 |
|  | Volume | 0.002 ± 0.0008 | 0.002 ± 0.0007 | -0.000151 | -0.641 | 0.26245 |


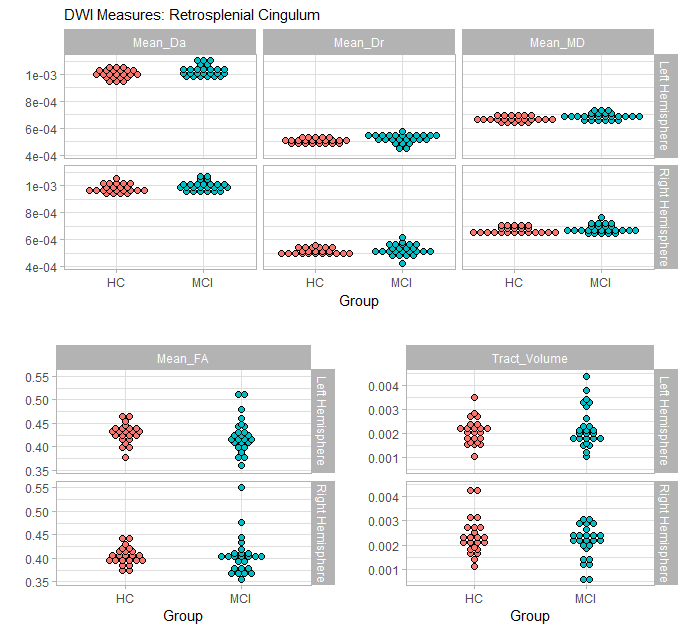


**S1 Fig C. Mean DWI measures of the retrosplenial cingulum**

**S1 Table E. Subgenual Cingulum** (P-value: uncorrected, 1-sided)

| Subgenual Cingulum | Measure | HC | MCI | Est | *t*-statistic | p-value |
| --- | --- | --- | --- | --- | --- | --- |
| Left | FA | 0.343 ± 0.037 | 0.329 ± 0.038 | -0.02407 | -2.173 | 0.01767 |
|  | MD | 0.00069 ± 0.00002 | 0.00070 ± 0.00002 | 0.00001516 | 3.078 | 0.00181 |
|  | Da | 0.00095 ± 0.00003 | 0.00096 ± 0.00004 | -0.00000201 | -0.215 | 0.41550 |
|  | Dr | 0.00055 ± 0.00003 | 0.00057 ± 0.00003 | 0.00002590 | 2.773 | 0.00409 |
|  | Volume | 0.002 ± 0.0007 | 0.002 ± 0.0007 | -0.0003431 | -1.850 | 0.03562 |
| Right | FA | 0.346 ± 0.037 | 0.350 ± 0.031 | -0.0003912 | -0.038 | 0.48505 |
|  | MD | 0.00069 ± 0.00002 | 0.00069 ± 0.00002 | 0.00000946 | 1.706 | 0.04765 |
|  | Da | 0.00095 ± 0.00003 | 0.00096 ± 0.00003 | 0.00000905 | 0.974 | 0.16770 |
|  | Dr | 0.00055 ± 0.00003 | 0.00055 ± 0.00003 | 0.00000655 | 0.687 | 0.24800 |
|  | Volume | 0.002 ± 0.0006 | 0.002 ± 0.0008 | 0.00000161 | 0.007 | 0.49750 |


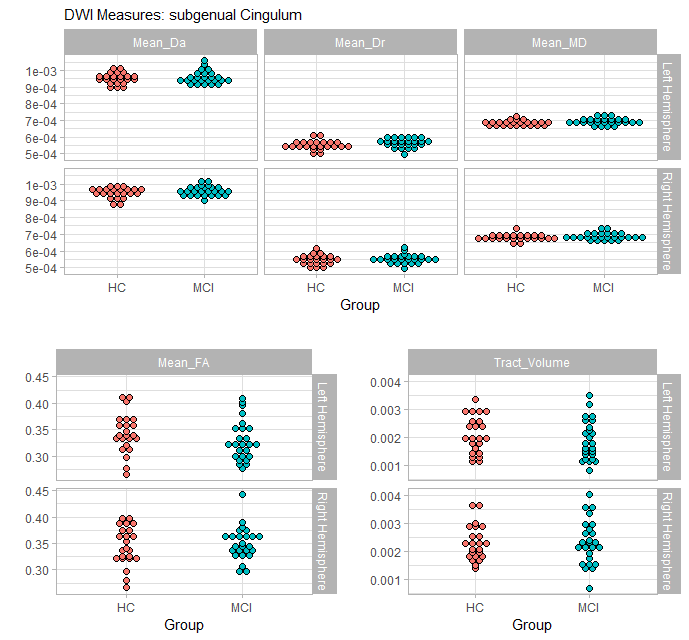


**S1 Fig D. Mean DWI measures of the subgenual cingulum**

**S1 Table F. Uncinate Fasciculus** (P-value: uncorrected, 1-sided)

| Uncinate Fasciculus | Measure | HC | MCI | Est | *t*-statistic | p-value |
| --- | --- | --- | --- | --- | --- | --- |
| Left | FA | 0.379 ± 0.029 | 0.359 ± 0.034 | -0.0118136 | -1.571 | 0.06150 |
|  | MD | 0.00070 ± 0.00002 | 0.00071 ± 0.00004 | 0.00000444 | 0.806 | 0.21225 |
|  | Da | 0.00101 ± 0.00003 | 0.00101 ± 0.00003 | -0.00000398 | -0.416 | 0.33970 |
|  | Dr | 0.00055 ± 0.00003 | 0.00057 ± 0.00004 | 0.00001122 | 1.734 | 0.04495 |
|  | Volume | 0.0006 ± 0.0005 | 0.0006 ± 0.0003 | 0.0000549 | 0.521 | 0.30250 |
| Right | FA | 0.381 ± 0.028 | 0.369 ± 0.037 | -0.0104457 | -1.483 | 0.07250 |
|  | MD | 0.00072 ± 0.00002 | 0.00072 ± 0.00003 | 0.00000254 | 0.336 | 0.36900 |
|  | Da | 0.00104 ± 0.00003 | 0.00103 ± 0.00003 | -0.00000784 | -0.808 | 0.2120 |
|  | Dr | 0.00055 ± 0.00003 | 0.00056 ± 0.00004 | 0.00000651 | 0.823 | 0.2075 |
|  | Volume | 0.001 ± 0.0007 | 0.0009 ± 0.0005 | -0.0002069 | -0.907 | 0.18450 |


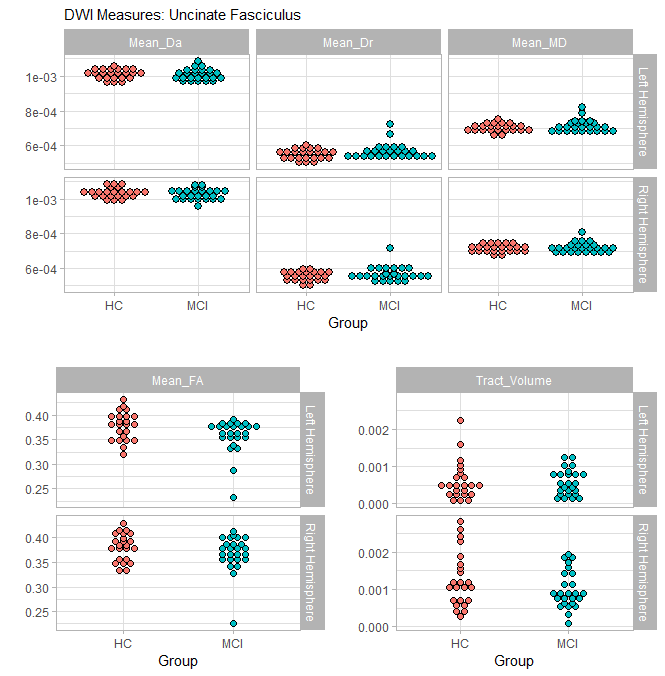


**S1 Fig E. Mean DWI measures of the uncinate fasciculus**

**Resting State MRI**

**S1 Table G. Between-network functional connectivity** (P-value: 2-sided uncorrected)

| Site / Hemisphere | HC | MCI | Estimate | *t*-statistic | P-value |
| --- | --- | --- | --- | --- | --- |
| **Left Hemisphere** |  |  |  |  |  |
| DMN -a & -b | 0.264 ± 0.093 | 0.206 ± 0.075 | -0.056598 | -2.240 | 0.01980 |
| DMN -a & -c | 0.352 ± 0.075 | 0.251 ± 0.111 | -0.107853 | -4.631 | 0.0000336 |
| DMN -b & -c | 0.064 ± 0.084 | 0.063 ± 0.068 | -0.010049 | -0.433 | 0.667 |
| DMN-a & Hippocampus | 0.238 ± 0.117 | 0.114 ± 0.125 | -0.149296 | -4.211 | 0.000127 |
| DMN-b & Hippocampus | 0.086 ± 0.117 | 0.103 ± 0.112 | 0.019833 | 0.550 | 0.585 |
| DMN-c & Hippocampus | 0.409 ± 0.096 | 0.262 ± 0.138 | -0.157262 | -3.988 | 0.000254 |
| DMN-a & Thalamus | 0.157 ± 0.097 | 0.108 ± 0.123 | -0.074604 | -2.153 | 0.0369 |
| DMN-b & Thalamus | 0.068 ± 0.089 | 0.087 ± 0.094 | 0.010637 | 0.360 | 0.721 |
| DMN-c & Thalamus | 0.182 ± 0.076 | 0.128 ± 0.139 | -0.055914 | -1.566 | 0.1247 |
| DMN-a & Limbic-a | 0.199 ± 0.084 | 0.164 ± 0.095 | -0.055350 | -2.028 | 0.0488 |
| DMN-b & Limbic-a | 0.099 ± 0.064 | 0.080 ± 0.077 | -0.030527 | -1.230 | 0.2253 |
| DMN-c & Limbic-a | 0.105 ± 0.104 | 0.082 ± 0.079 | -0.054109 | -2.481 | 0.01709 |
| DMN-a & Limbic-b | 0.055 ± 0.063 | 0.042 ± 0.076 | -0.021438 | -1.090 | 0.2820 |
| DMN-b & Limbic-b | 0.084 ± 0.062 | 0.085 ± 0.059 | -0.013014 | -0.865 | 0.3917 |
| DMN-c & Limbic-b | 0.064 ± 0.066 | 0.096 ± 0.058 | 0.024549 | 1.314 | 0.19597 |
| Limbic-a & Hippocampus | 0.025 ± 0.128 | 0.035 ± 0.123 | 0.004507 | 0.117 | 0.908 |
| Limbic-a & Thalamus | 0.009 ± 0.139 | 0.008 ± 0.115 | -0.037312 | -1.037 | 0.3054 |
| Limbic-b & Hippocampus | 0.141 ± 0.118 | 0.122 ± 0.102 | -0.031263 | -0.887 | 0.380 |
| Limbic-b & Thalamus | -0.009 ± 0.115 | -0.017 ± 0.106 | -0.034707 | -1.115 | 0.2711 |
| Limbic-a & Limbic-b | 0.022 ± 0.092 | 0.054 ± 0.102 | 0.025764 | 0.747 | 0.459 |
| Hippocampus & Thalamus | 0.256 ± 0.128 | 0.301 ± 0.248 | 0.032212 | 0.503 | 0.618 |
| **Right Hemisphere** |  |  |  |  |  |
| DMN -a & -b | 0.238 ± 0.083 | 0.211 ± 0.095 | -0.055302 | -2.358 | 0.022990 |
| DMN -a & -c | 0.328 ± 0.089 | 0.217 ± 0.092 | -0.116434 | -3.809 | 0.000438 |
| DMN -b & -c | 0.046 ± 0.071 | 0.036 ± 0.082 | -0.034413 | -1.754 | 0.08561 |
| DMN-a & Hippocampus | 0.208 ± 0.123 | 0.104 ± 0.156 | -0.128291 | -3.306 | 0.00192 |
| DMN-b & Hippocampus | 0.022 ± 0.133 | 0.091 ± 0.098 | 0.060454 | 1.892 | 0.0652 |
| DMN-c & Hippocampus | 0.326 ± 0.124 | 0.186 ± 0.137 | -0.157239 | -3.524 | 0.00102 |
| DMN-a & Thalamus | 0.137 ± 0.084 | 0.091 ± 0.108 | -0.054046 | -1.994 | 0.0525 |
| DMN-b & Thalamus | 0.043 ± 0.095 | 0.054 ± 0.088 | -0.005408 | -0.191 | 0.8492 |
| DMN-c & Thalamus | 0.147 ± 0.091 | 0.039 ± 0.144 | -0.10986 | -3.023 | 0.00408 |
| DMN-a & Limbic-a | 0.167 ± 0.078 | 0.146 ± 0.064 | -0.042455 | -1.971 | 0.05521 |
| DMN-b & Limbic-a | 0.085 ± 0.055 | 0.112 ± 0.061 | 0.016385 | 0.875 | 0.3863 |
| DMN-c & Limbic-a | 0.133 ± 0.085 | 0.080 ± 0.069 | -0.081981 | -4.007 | 0.00024 |
| DMN-a & Limbic-b | 0.114 ± 0.065 | 0.104 ± 0.077 | -0.030324 | -1.677 | 0.10082 |
| DMN-b & Limbic-b | 0.114 ± 0.055 | 0.123 ± 0.051 | 0.000994 | 0.066 | 0.94797 |
| DMN- C & Limbic-b | 0.115 ± 0.078 | 0.113 ± 0.069 | -0.020170 | -1.076 | 0.2878 |
| Limbic-a & Hippocampus | 0.099 ± 0.128 | 0.069 ± 0.127 | -0.051616 | -1.159 | 0.253 |
| Limbic-a & Thalamus | 0.026 ± 0.106 | 0.016 ± 0.112 | -0.049676 | -1.873 | 0.0678 |
| Limbic-a & Hippocampus | 0.101 ± 0.097 | 0.115 ± 0.127 | 0.004334 | 0.146 | 0.8850 |
| Limbic-b & Thalamus | -0.030 ± 0.108 | -0.049 ± 0.119 | -0.032760 | -0.959 | 0.343 |
| Limbic-a & Limbic-b | 0.043 ± 0.084 | 0.092 ± 0.094 | 0.037690 | 1.429 | 0.1603 |
| Hippocampus & Thalamus | 0.260 ± 0.145 | 0.201 ± 0.194 | -0.032684 | -0.642 | 0.525 |

**S1 Fig F: Correlation between Fornix mean diffusivity and within-network functional connecitivity**


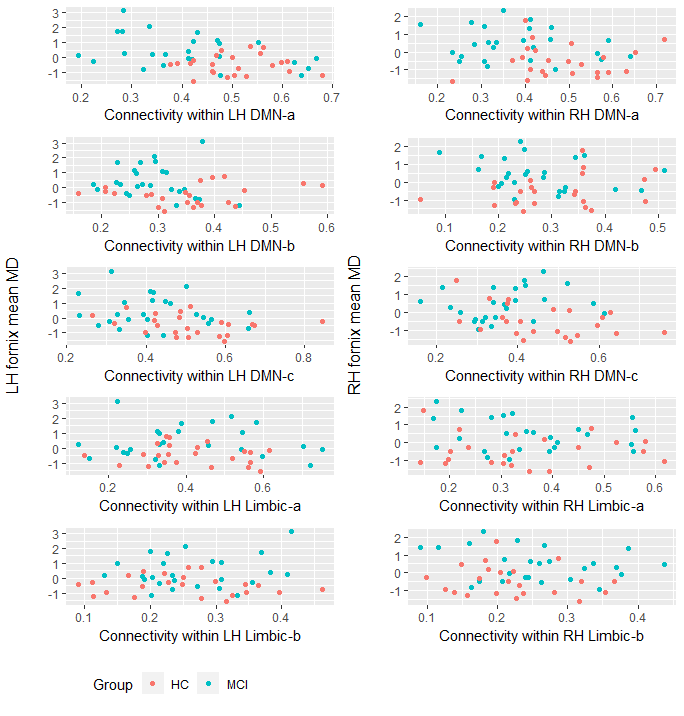


| LH/group | Robust Correlation  Coefficient | Test Statistic | p-value uncor | RH/group | Robust Correlation  Coefficient | Test Statistic | p-value uncor |
| --- | --- | --- | --- | --- | --- | --- | --- |
| **MCI** |  | | | | | | |
| DMN-a | -0.347 | -1.76 | 0.093 | DMN-a | -0.069 | -0.332 | 0.743 |
| DMN-b | -0.248 | -1.23 | 0.231 | DMN-b | -0.283 | -1.42 | 0.170 |
| DMN-c | -0.116 | -0.562 | 0.580 | DMN-c | 0.333 | 1.69 | 0.104 |
| Limbic-a | 0.105 | 0.505 | 0.619 | Limbic-a | -0.190 | -0.929 | 0.362 |
| Limbic-b | 0.078 | 0.376 | 0.710 | Limbic-b | -0.206 | -1.01 | 0.324 |
| **HC** |  | | | | | | |
| DMN-a | 0.052 | 0.239 | 0.813 | DMN-a | -0.016 | -0.073 | 0.943 |
| DMN-b | 0.143 | 0.662 | 0.515 | DMN-b | 0.236 | 1.11 | 0.279 |
| DMN-c | -0.323 | -1.57 | 0.133 | DMN-c | -0.335 | -1.63 | 0.119 |
| Limbic-a | -0.141 | -0.654 | 0.521 | Limbic-a | 0.069 | 0.316 | 0.755 |
| Limbic-b | -0.156 | -0.722 | 0.478 | Limbic-b | -0.281 | -1.34 | 0.194 |

**S1 Fig G. Correlation of parahippocampal cingulum MD & within-network functional connecitivity**


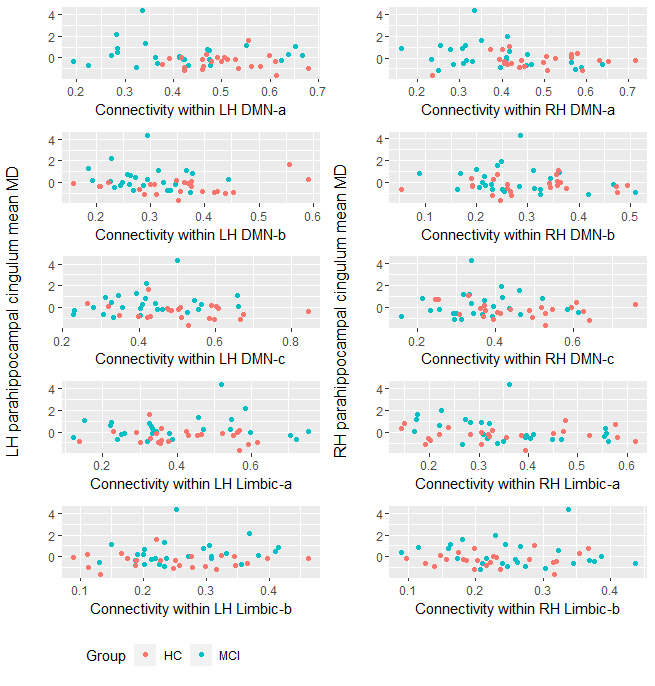


| LH/group | Robust Correlation  Coefficient | Test Statistic | p-value  uncor | RH/group | Robust Correlation  Coefficient | Test Statistic | p-value uncor |
| --- | --- | --- | --- | --- | --- | --- | --- |
| **MCI** |  | | | | | | |
| DMN-a | 0.096 | 0.464 | 0.647 | DMN-a | -0.371 | -1.92 | 0.068 |
| DMN-b | -0.133 | -0.641 | 0.528 | DMN-b | -0.243 | -1.20 | 0.242 |
| DMN-c | 0.226 | 1.11 | 0.278 | DMN-c | 0.193 | 0.944 | 0.355 |
| Limbic-a | 0.082 | 0.396 | 0.696 | Limbic-a | -0.525 | -2.96 | 0.007 |
| Limbic-b | 0.227 | 1.12 | 0.276 | Limbic-b | -0.327 | -1.66 | 0.110 |
| **HC** |  | | | | | | |
| DMN-a | -0.057 | -0.263 | 0.796 | DMN-a | 0.004 | 0.017 | 0.986 |
| DMN-b | -0.082 | -0.356 | 0.711 | DMN-b | 0.126 | 0.583 | 0.566 |
| DMN-c | -0.177 | -0.823 | 0.420 | DMN-c | -0.228 | -1.07 | 0.295 |
| Limbic-a | -0.072 | -0.330 | 0.744 | Limbic-a | 0.042 | 0.191 | 0.851 |
| Limbic-b | -0.033 | -0.50 | 0.882 | Limbic-b | 0.112 | 0.516 | 0.611 |

**S1 Fig H. Correlation of retrosplenial cingulum MD & within-network functional connecitivity**


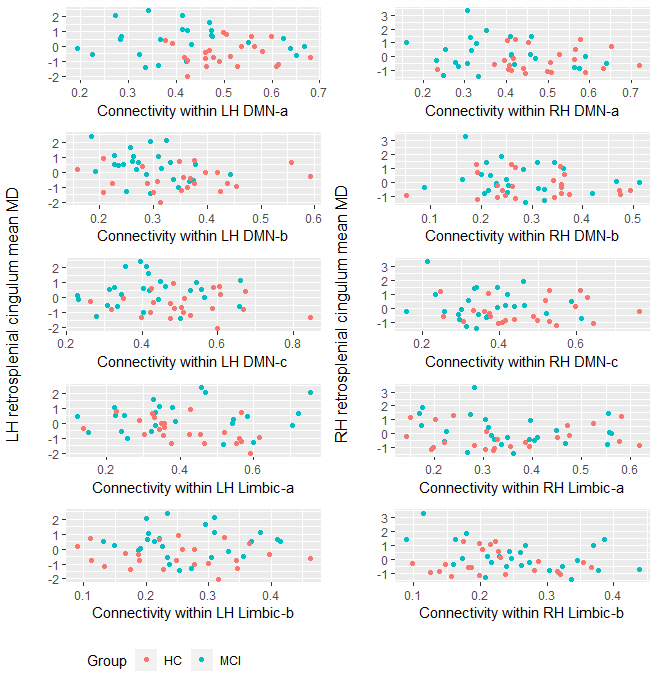


| LH/group | Robust Correlation  Coefficient | Test Statistic | p-value uncor | RH/group | Robust Correlation  Coefficient | Test Statistic | p-value uncor |
| --- | --- | --- | --- | --- | --- | --- | --- |
| **MCI** |  | | | | | | |
| DMN-a | -0.020 | -0.094 | 0.926 | DMN-a | -0.090 | -0.435 | 0.668 |
| DMN-b | -0.341 | -1.74 | 0.095 | DMN-b | -0.096 | -0.462 | 0.648 |
| DMN-c | 0.122 | 0.594 | 0.559 | DMN-c | 0.032 | 0.154 | 0.879 |
| Limbic-a | 0.073 | 0.350 | 0.730 | Limbic-a | -0.286 | -1.432 | 0.166 |
| Limbic-b | 0.029 | 0.141 | 0.890 | Limbic-b | -0.362 | -1.860 | 0.076 |
| **HC** |  | | | | | | |
| DMN-a | 0.042 | 0.195 | 0.848 | DMN-a | 0.137 | 0.633 | 0.534 |
| DMN-b | 0.50 | 0.229 | 0.821 | DMN-b | -0.080 | -0.367 | 0.717 |
| DMN-c | 0.041 | 0.189 | 0.852 | DMN-c | 0.134 | 0.619 | 0.543 |
| Limbic-a | -0.524 | -2.82 | 0.010 | Limbic-a | 0.076 | 0.347 | 0.732 |
| Limbic-b | -0.055 | -0.252 | 0.803 | Limbic-b | -0.112 | -0.518 | 0.610 |

**S1 Fig I: Correlation of subgenual cingulum MD & within-network functional connecitivity**


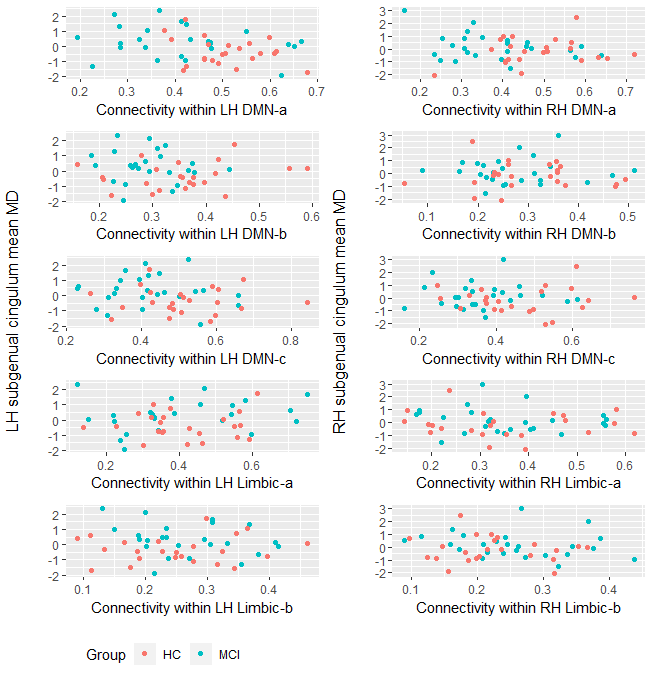


| LH/group | Robust Correlation  Coefficient | Test Statistic | p-value uncor | RH/group | Robust Correlation  Coefficient | Test Statistic | p-value uncor |
| --- | --- | --- | --- | --- | --- | --- | --- |
| **MCI** |  | | | | | | |
| DMN-a | -0.149 | -0.720 | 0.479 | DMN-a | -0.158 | -0.768 | 0.450 |
| DMN-b | -0.140 | -0.677 | 0.505 | DMN-b | -0.088 | -0.423 | 0.676 |
| DMN-c | -0.039 | -0.187 | 0.854 | DMN-c | 0.106 | 0.0510 | 0.615 |
| Limbic-a | 0.288 | 1.44 | 0.163 | Limbic-a | -0.264 | -1.31 | 0.202 |
| Limbic-b | -0.227 | -1.12 | 0.274 | Limbic-b | -0.291 | -1.46 | 0.158 |
| **HC** |  | | | | | | |
| DMN-a | -0.124 | -0.574 | 0.572 | DMN-a | 0.065 | 0.301 | 0.767 |
| DMN-b | 0.210 | 0.986 | 0.336 | DMN-b | 0.019 | 0.089 | 0.931 |
| DMN-c | 0.018 | 0.081 | 0.936 | DMN-c | -0.008 | -0.038 | 0.970 |
| Limbic-a | -0.040 | -0.184 | 0.856 | Limbic-a | -0.011 | -0.052 | 0.959 |
| Limbic-b | 0.050 | 0.231 | 0.820 | Limbic-b | -0.046 | -0.212 | 0.834 |

**S1 Figure J. Correlation of uncinate fasciculus MD & within-network functional connecitivity**


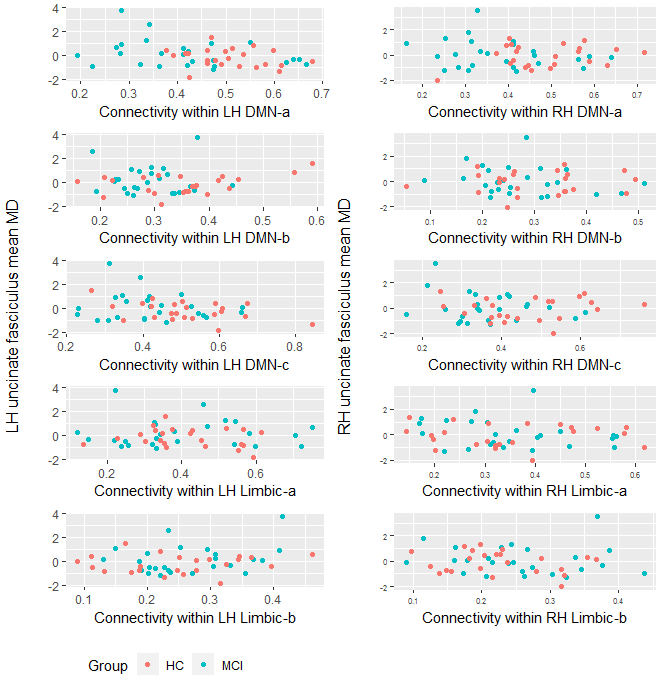


| LH/group | Robust Correlation  Coefficient | Test Statistic | p-value uncor | RH/group | Robust Correlation  Coefficient | Test Statistic | p-value uncor |
| --- | --- | --- | --- | --- | --- | --- | --- |
| **MCI** |  | | | | | | |
| DMN-a | -0.267 | -1.33 | 0.196 | DMN-a | -0.208 | -1.02 | 0.318 |
| DMN-b | -0.088 | -0.422 | 0.677 | DMN-b | -0.228 | -1.12 | 0.273 |
| DMN-c | -0.089 | -0.429 | 0.672 | DMN-c | -0.045 | -0.217 | 0.830 |
| Limbic-a | 0.126 | 0.611 | 0.547 | Limbic-a | -0.331 | -1.68 | 0.107 |
| Limbic-b | 0.187 | 0.911 | 0.372 | Limbic-b | -0.157 | -0.764 | 0.453 |
| **HC** |  | | | | | | |
| DMN-a | -0.292 | -1.40 | 0.176 | DMN-a | 0.094 | 0.432 | 0.670 |
| DMN-b | 0.183 | 0.854 | 0.403 | DMN-b | -0.019 | -0.087 | 0.931 |
| DMN-c | -0.238 | -1.12 | 0.275 | DMN-c | 0.178 | 0.829 | 0.416 |
| Limbic-a | -0.140 | -0.648 | 0.524 | Limbic-a | 0.077 | 0.355 | 0.726 |
| Limbic-b | 0.212 | 0.995 | 0.331 | Limbic-b | -0.174 | -0.811 | 0.427 |

**S1 Table H. Graph theory cluster coefficient, sparsity level 0.10**

| **Measure** | **HC** | **MCI** | **Estimate** | ***t*-statistic** | **P-value*** |
| --- | --- | --- | --- | --- | --- |
| **LH** |  |  |  |  |  |
| DMN-b Temp 3 | 0.558 ± 0.058 | 0.460 ± 0.100 | 0.10 | -4.25 | 0.000114 |
| DMN-a PCC 1 | 0.575 ± 0.079 | 0.490 ± 0.082 | 0.10 | -4.14 | 0.000161 |
| DMN-a PCC 2 | 0.555 ± 0.054 | 0.496 ± 0.075 | 0.07 | -3.70 | 0.000608 |
| SomMot A 16 | 0.531 ± 0.0 | 0.423 ± 0.094 | 0.11 | -3.60 | 0.000808 |
| DMN-b PFCd 1 | 0.530 ± 0.071 | 0.453 ± 0.084 | 0.08 | -3.58 | 0.000871 |
| Hippocampus | 0.498 ± 0.126 | 0.369 ± 0.132 | 0.14 | -3.49 | 0.001137 |
| DMN-a PFCm 3 | 0.561 ± 0.077 | 0.483 ± 0.094 | 0.09 | -3.47 | 0.001198 |
| DMN-b Temp 2 | 0.568 ± 0.089 | 0.478 ± 0.097 | 0.09 | -3.33 | 0.001781 |
| **RH** |  |  |  |  |  |
| Hippocampus | 0.527 ± 0.125 | 0.357 ± 0.136 | 0.20 | -5.53 | 0.000002 |
| DMN-a PFCm 1 | 0.536 ± 0.068 | 0.450 ± 0.080 | 0.09 | -4.12 | 0.000170 |
| DMN-b PFCd 4 | 0.555 ± 0.076 | 0.475 ± 0.107 | 0.10 | -3.72 | 0.000572 |
| Limibic-a OFC 6 | 0.553 ± 0.079 | 0.453 ± 0.112 | 0.11 | -3.60 | 0.000818 |
| SalVentAttn A ParMed 8 | 0.500 ± 0.101 | 0.426 ± 0.102 | 0.10 | -3.49 | 0.001124 |
| SalVentAttn A ParMed 4 | 0.487 ± 0.094 | 0.399 ± 0.090 | 0.10 | -3.46 | 0.001235 |
| DMN-a PFCd 2 | 0.555 ± 0.078 | 0.463 ± 0.095 | 0.09 | -3.45 | 0.001268 |
| DMN-a PCC 1 | 0.559 ± 0.064 | 0.494 ± 0.077 | 0.07 | -3.36 | 0.001649 |

** p*-values are uncorrected 2-sided

Graph theory analysis, at sparsity level 0.10, revealed a significant between-group difference in the cluster coefficient measure. This was higher in the HC (M=0.484, SD=0.027) compared to the MCI (M=0.461, SD=0.035) group (**b** = .03, *t* = -2.90, *p*_uncor_ = .006). See table H above.

Graph theory analysis, at sparsity level 0.20, revealed a significant between-group difference in the cluster coefficient measure. This was higher in the HC (M=0.504, SD=0.025) compared to the MCI (M=0.490, SD=0.031) group (**b** = .02, *t* = -2.31, *p*_uncor_ = .026). The ROIs driving this difference did not survive correction for multiple comparisons.

Graph theory analysis, at sparsity level 0.25, revealed a significant between-group difference in the cluster coefficient measure. This was higher in the HC (M=0.521, SD=0.024) compared to the MCI (M=0.509, SD=0.029) group (**b** = .02, *t* = -2.19, *p*_uncor_ = .034). See table I.

**S1 Table I. Graph theory cluster coefficient, sparsity level 0.25**

| **Measure** | **HC** | **MCI** | **Estimate** | ***t*-statistic** | **P-value*** |
| --- | --- | --- | --- | --- | --- |
| **LH** |  |  |  |  |  |
| DMN-b Temp 3 | 0.580 ± 0.051 | 0.512 ± 0.070 | 0.08 | -4.69 | 0.00028 |

** p*-values are uncorrected 2-sided

Graph theory analysis, at sparsity level 0.10, revealed a statistical difference in average path length between the groups, with a longer path length in HC compared to controls (*b* = .05, *t* = -2.42, *p*_uncor_ = .020; HC: M = 2.39, SD = 0.071; MCI: M = 2.35, SD = 0.084). This difference was driven by two ROIs in the somatomotor network - one in the left hemisphere and one in the right.

**S1 Table J. Graph theory average path length, sparsity level 0.10**

| **Measure** | **HC** | **MCI** | **Estimate** | ***t*-statistic** | **P-value*** |
| --- | --- | --- | --- | --- | --- |
| **LH** |  |  |  |  |  |
| SomMot-a 15 | 2.46 ± 0.215 | 2.22. ± 0.180 | 0.25 | -4.25 | 0.000112 |
| **RH** |  |  |  |  |  |
| SomMot-a 13 | 2.45 ± 0.129 | 2.25 ± 0.200 | 0.19 | -4.20 | 0.000133 |

** p*-values are uncorrected 2-sided

Graph theory analysis did not reveal a stastical difference in average path length between the two groups at sparsity level 0.20: (*b* = .02, *t* = -1.54, *p*_uncor_ = .130; HC: M = 1.89, SD = 0.035; MCI: M = 1.88, SD = 0.040), nor at sparsity level 0.25: (*b* = .01, *t* = 1.11, *p*_uncor_ = .275; HC: M = 1.78, SD = 0.024; MCI: M = 1.78, SD = 0.029).

FSL-ANAT segmentation method was used to extract tissue volumes (grey matter, white matter and cerebrospinal fluid). Partial volume tissue estimates were used in native space to calculate intrac-cranial volume. DWI tract volumes were divided by intracranial volume to provide comparable tract volumes for the DWI analysis. Tissue estimates were converted to MNI space for the below commparison.


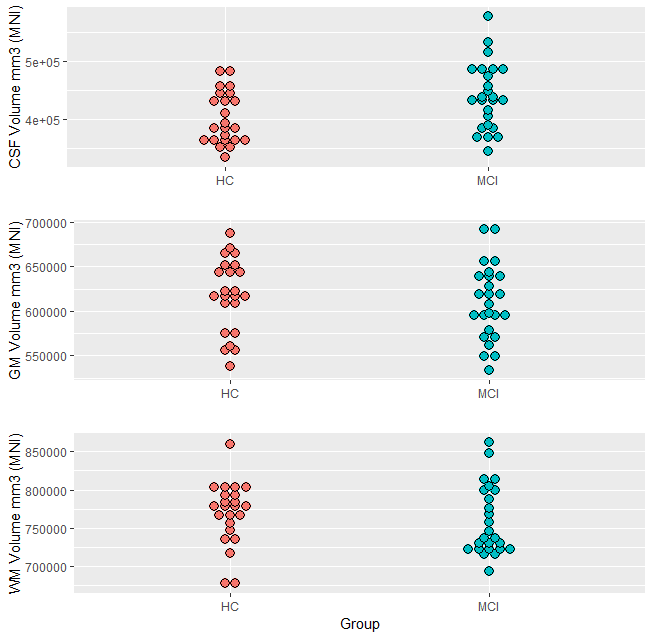


**S1 Fig K. Partial Volume estimates of whole brain tissue type in MNI space**

**S1 Table K. Tissue volumes** (mean ± SD), one-sided uncorrected p-value from linear model inc covariates

| Measure | HC | MCI | *t*-statistic | p-value |
| --- | --- | --- | --- | --- |
| CSF | 402588 ± 44821 | 440354 ± 57147 | -2.87 | .003 |
| Grey matter | 618049 ± 41310 | 609618 ± 42238 | 2.67 | .005 |
| White matter | 769028 ± 41357 | 759327 ± 45236 | 2.05 | .023 |

**S1 Table L. Schaefer atlas 2018 400 parcels 17 networks MNI152 2mm – centroid co-ordinates for default mode and limbic networks.** Elements shaded in grey were implicated in one/more graph theory analyses. For complete list of centroids see: https://github.com/ThomasYeoLab/CBIG/blob/master/stable_projects/brain_parcellation/Schaefer2018_LocalGlobal/Parcellations/MNI/Centroid_coordinates/Schaefer2018_400Parcels_17Networks_order_FSLMNI152_2mm.Centroid_RAS.csv

| **ROI Index** | **Label Name** | **X** | **Y** | **Z** |
| --- | --- | --- | --- | --- |
| 36 | 17Networks_LH_SomMotA_12 | -24 | -10 | 64 |
| 39 | 17Networks_LH_SomMotA_15 | -4 | -26 | 68 |
| 40 | 17Networks_LH_SomMotA_16 | -14 | -12 | 72 |
| 109 | 17Networks_LH_Limbic_OFC_1 | -12 | 24 | -20 |
| 110 | 17Networks_LH_Limbic_OFC_2 | -24 | 22 | -20 |
| 111 | 17Networks_LH_Limbic_OFC_3 | -10 | 48 | -22 |
| 112 | 17Networks_LH_Limbic_OFC_4 | -4 | 24 | -20 |
| 113 | 17Networks_LH_Limbic_OFC_5 | -16 | 64 | -8 |
| 114 | 17Networks_LH_Limbic_TempPole_1 | -38 | -6 | -42 |
| 115 | 17Networks_LH_Limbic_TempPole_2 | -24 | 6 | -40 |
| 116 | 17Networks_LH_Limbic_TempPole_3 | -26 | -10 | -32 |
| 117 | 17Networks_LH_Limbic_TempPole_4 | -54 | -22 | -30 |
| 118 | 17Networks_LH_Limbic_TempPole_5 | -40 | -22 | -26 |
| 119 | 17Networks_LH_Limbic_TempPole_6 | -32 | 12 | -30 |
| 120 | 17Networks_LH_Limbic_TempPole_7 | -44 | 6 | -16 |
| 149 | 17Networks_LH_DefaultA_IPL_1 | -48 | -64 | 32 |
| 150 | 17Networks_LH_DefaultA_IPL_2 | -42 | -72 | 44 |
| 151 | 17Networks_LH_DefaultA_PFCd_1 | -24 | 28 | 44 |
| 152 | 17Networks_LH_DefaultA_PFCd_2 | -18 | 36 | 48 |
| 153 | 17Networks_LH_DefaultA_PFCd_3 | -22 | 20 | 52 |
| 154 | 17Networks_LH_DefaultA_PCC_1 | -4 | -54 | 20 |
| 155 | 17Networks_LH_DefaultA_PCC_2 | -6 | -60 | 30 |
| 156 | 17Networks_LH_DefaultA_PCC_3 | -8 | -44 | 32 |
| 157 | 17Networks_LH_DefaultA_PCC_4 | -4 | -34 | 38 |
| 158 | 17Networks_LH_DefaultA_PCC_5 | -2 | -16 | 38 |
| 159 | 17Networks_LH_DefaultA_PCC_6 | -2 | -68 | 42 |
| 160 | 17Networks_LH_DefaultA_PCC_7 | -6 | -50 | 42 |
| 161 | 17Networks_LH_DefaultA_PFCm_1 | -4 | 56 | -10 |
| 162 | 17Networks_LH_DefaultA_PFCm_2 | -6 | 36 | -8 |
| 163 | 17Networks_LH_DefaultA_PFCm_3 | -6 | 60 | 6 |
| 164 | 17Networks_LH_DefaultA_PFCm_4 | -6 | 44 | 6 |
| 165 | 17Networks_LH_DefaultA_PFCm_5 | -16 | 68 | 8 |
| 166 | 17Networks_LH_DefaultA_PFCm_6 | -6 | 34 | 20 |
| 167 | 17Networks_LH_DefaultB_Temp_1 | -44 | 12 | -34 |
| 168 | 17Networks_LH_DefaultB_Temp_2 | -54 | -2 | -30 |
| 169 | 17Networks_LH_DefaultB_Temp_3 | -62 | -18 | -20 |
| 170 | 17Networks_LH_DefaultB_Temp_4 | -56 | -8 | -14 |
| 171 | 17Networks_LH_DefaultB_Temp_5 | -60 | -34 | -4 |
| 172 | 17Networks_LH_DefaultB_Temp_6 | -52 | -22 | -6 |
| 173 | 17Networks_LH_DefaultB_IPL_1 | -46 | -58 | 20 |
| 174 | 17Networks_LH_DefaultB_IPL_2 | -56 | -54 | 30 |
| 175 | 17Networks_LH_DefaultB_PFCd_1 | -4 | 52 | 28 |
| 176 | 17Networks_LH_DefaultB_PFCd_2 | -14 | 58 | 30 |
| 177 | 17Networks_LH_DefaultB_PFCd_3 | -22 | 50 | 32 |
| 178 | 17Networks_LH_DefaultB_PFCd_4 | -8 | 42 | 52 |
| 179 | 17Networks_LH_DefaultB_PFCd_5 | -12 | 24 | 60 |
| 180 | 17Networks_LH_DefaultB_PFCd_6 | -6 | 10 | 64 |
| 181 | 17Networks_LH_DefaultB_PFCl_1 | -40 | 20 | 48 |
| 182 | 17Networks_LH_DefaultB_PFCl_2 | -42 | 8 | 48 |
| 183 | 17Networks_LH_DefaultB_PFCv_1 | -36 | 22 | -16 |
| 184 | 17Networks_LH_DefaultB_PFCv_2 | -36 | 36 | -12 |
| 185 | 17Networks_LH_DefaultB_PFCv_3 | -46 | 32 | -10 |
| 186 | 17Networks_LH_DefaultB_PFCv_4 | -48 | 28 | 0 |
| 187 | 17Networks_LH_DefaultB_PFCv_5 | -54 | 20 | 12 |
| 188 | 17Networks_LH_DefaultC_IPL_1 | -40 | -78 | 30 |
| 189 | 17Networks_LH_DefaultC_Rsp_1 | -14 | -48 | 4 |
| 190 | 17Networks_LH_DefaultC_Rsp_2 | -8 | -52 | 10 |
| 191 | 17Networks_LH_DefaultC_Rsp_3 | -14 | -60 | 18 |
| 192 | 17Networks_LH_DefaultC_PHC_1 | -20 | -20 | -26 |
| 193 | 17Networks_LH_DefaultC_PHC_2 | -30 | -32 | -18 |
| 194 | 17Networks_LH_DefaultC_PHC_3 | -18 | -38 | -12 |
| 236 | 17Networks_RH_SomMotA_13 | 10 | -40 | 68 |
| 299 | 17Networks_RH_SalVentAttnA_ParMed_4 | 6 | 10 | 58 |
| 302 | 17Networks_RH_SalVentAttnA_ParMed_7 | 6 | -2 | 66 |
| 303 | 17Networks_RH_SalVentAttnA_ParMed_8 | 16 | 6 | 70 |
| 313 | 17Networks_RH_Limbic_OFC_1 | 14 | 24 | -20 |
| 314 | 17Networks_RH_Limbic_OFC_2 | 22 | 22 | -20 |
| 315 | 17Networks_RH_Limbic_OFC_3 | 8 | 46 | -24 |
| 316 | 17Networks_RH_Limbic_OFC_4 | 20 | 42 | -18 |
| 317 | 17Networks_RH_Limbic_OFC_5 | 4 | 22 | -20 |
| 318 | 17Networks_RH_Limbic_OFC_6 | 10 | 62 | -14 |
| 319 | 17Networks_RH_Limbic_TempPole_1 | 28 | -2 | -40 |
| 320 | 17Networks_RH_Limbic_TempPole_2 | 48 | -6 | -40 |
| 321 | 17Networks_RH_Limbic_TempPole_3 | 36 | 18 | -38 |
| 322 | 17Networks_RH_Limbic_TempPole_4 | 40 | -14 | -32 |
| 323 | 17Networks_RH_Limbic_TempPole_5 | 28 | 12 | -30 |
| 324 | 17Networks_RH_Limbic_TempPole_6 | 50 | -28 | -26 |
| 358 | 17Networks_RH_DefaultA_Temp_1 | 60 | -8 | -24 |
| 359 | 17Networks_RH_DefaultA_IPL_1 | 54 | -54 | 26 |
| 360 | 17Networks_RH_DefaultA_IPL_2 | 48 | -64 | 42 |
| 361 | 17Networks_RH_DefaultA_PFCd_1 | 26 | 34 | 38 |
| 362 | 17Networks_RH_DefaultA_PFCd_2 | 24 | 26 | 50 |
| 363 | 17Networks_RH_DefaultA_PCC_1 | 6 | -52 | 24 |
| 364 | 17Networks_RH_DefaultA_PCC_2 | 4 | -64 | 32 |
| 365 | 17Networks_RH_DefaultA_PCC_3 | 6 | -38 | 34 |
| 366 | 17Networks_RH_DefaultA_PCC_4 | 4 | -20 | 36 |
| 367 | 17Networks_RH_DefaultA_PCC_5 | 10 | -52 | 36 |
| 368 | 17Networks_RH_DefaultA_PFCm_1 | 6 | 42 | -10 |
| 369 | 17Networks_RH_DefaultA_PFCm_2 | 10 | 66 | 0 |
| 370 | 17Networks_RH_DefaultA_PFCm_3 | 8 | 42 | 4 |
| 371 | 17Networks_RH_DefaultA_PFCm_4 | 8 | 54 | 12 |
| 372 | 17Networks_RH_DefaultA_PFCm_5 | 18 | 64 | 16 |
| 373 | 17Networks_RH_DefaultA_PFCm_6 | 6 | 26 | 18 |
| 374 | 17Networks_RH_DefaultB_Temp_1 | 64 | -24 | -8 |
| 375 | 17Networks_RH_DefaultB_Temp_2 | 64 | -38 | 0 |
| 376 | 17Networks_RH_DefaultB_AntTemp_1 | 50 | 8 | -32 |
| 377 | 17Networks_RH_DefaultB_PFCd_1 | 6 | 58 | 30 |
| 378 | 17Networks_RH_DefaultB_PFCd_2 | 16 | 52 | 36 |
| 379 | 17Networks_RH_DefaultB_PFCd_3 | 4 | 44 | 40 |
| 380 | 17Networks_RH_DefaultB_PFCd_4 | 14 | 38 | 52 |
| 381 | 17Networks_RH_DefaultB_PFCd_5 | 12 | 20 | 62 |
| 382 | 17Networks_RH_DefaultB_PFCv_1 | 34 | 22 | -18 |
| 383 | 17Networks_RH_DefaultB_PFCv_2 | 48 | 32 | -8 |
| 384 | 17Networks_RH_DefaultB_PFCv_3 | 54 | 24 | 6 |
| 385 | 17Networks_RH_DefaultC_IPL_1 | 48 | -64 | 22 |
| 386 | 17Networks_RH_DefaultC_IPL_2 | 46 | -76 | 30 |
| 387 | 17Networks_RH_DefaultC_Rsp_1 | 14 | -46 | 4 |
| 388 | 17Networks_RH_DefaultC_Rsp_2 | 12 | -56 | 16 |
| 389 | 17Networks_RH_DefaultC_PHC_1 | 22 | -18 | -28 |
| 390 | 17Networks_RH_DefaultC_PHC_2 | 30 | -30 | -18 |
