# Supplementary material for: No relationship between fornix and cingulum degradation and within-network decreases in functional connectivity in prodromal Alzheimer’s disease
Source: PLoS One. 2019 Oct 3;14(10):e0222977. doi: 10.1371/journal.pone.0222977 (PMC6776361; doi:10.1371/journal.pone.0222977)
Supplement: Attachment — Submitted filename: Response to Reviewers.docx [file pone.0222977.s002.docx]

**Reviewer #1:**

**This paper investigated changes in functional and structural connectivity among regions that support memory function in preclinical Alzheimer’s disease. Then investigated the relationship between white matter tracts (fornix, cingulum bundles, and uncinate fasciculus) and functional connectivity. It’s very interesting and meaningful. But there are some issues to be resolved as following.**

Overall Reply: We thank the reviewer for their comments, and their appreciation and help in producing the improvements made to the manuscript.

**Main concerns:**
**1. L313: “Cost (sparsity) was set at 0.15”. How about other costs? Are the results similar or seriously different?**

Thank you for this feedback. Given our start of 0.15 we chose to investigate this further using the additional levels: 0.10, 0.20, and 0.25.

We have inserted the following text within the results section:

Given the challenges of defining a-priori an appropriate network cost level (94), and following feedback, we additionally inspected the graph theory measures at sparsity levels of .10, .20 and .25. There were significant differences between the two groups in the cluster coefficient measure for sparsity levels of 0.10, 0.20 and 0.25 (uncorrected for multiple comparisons across levels). As per cost level 0.15, the cluster coefficient measures were higher in the HC compared to the MCI group. At level 0.10 the difference was driven by 16 ROIs (12 from the DMN and hippocampus – 10 of which drive the group differences at sparsity level 0.15). At level 0.20 no individual ROI survived correction for multiple comparisons. At level 0.25 the difference was driven exclusively by LH temporal area 3 of the DMN_b (it was also a driver of differences at levels 0.10 and 0.15). Full details are provided in S1 file. There was a significant difference in the average path length between the groups at sparsity level of 0.10. As per sparsity level 0.15, this average path length difference was not present at levels 0.20 and 0.25. Further details are provided in S1 file.

We have added the below highlighted piece of text in the results section:

The whole-brain graph theory measures revealed that the areas of difference between the two groups were centred on DMN nodes and the hippocampus. The analysis of the cluster coefficient (how well specialist information is segregated) showed that across both hemispheres there were fewer connections-between-nearest-neighbours of select DMN-a and DMN-b nodes and of the hippocampus in the MCI group, at cost levels of 0.10 and 0.15. This metric, related to the resilience of local networks, suggests that these areas in the MCI group are relatively more exposed to insult (114,115). The presence of the hippocampus and DMN-a in the results from both analyses (functional connectivity strength and graph theory) may indicate that it has both lost connections and that its remaining connections are also weaker.

We have added the following highlighted pieces of text to the Limitations section:

Constraints regarding the interpretation of graph theory analysis include those mentioned above for rsMRI (excluding the region of interest approach) and are extended by its binarisation process (119). In order to achieve a high-level overview, it meant that in this instance, only a restricted range of cost % of connections based on correlation strength were included in the analysis. With this reductive approach valuable information is lost and the risk of a skewed understanding of clinically important brain connectivity differences is increased (118). It should also be noted that a widespread difference in underlying functional connectivity between patient and control groups may introduce potential artifactual differences in network topology metrics (120).

We have inserted the following details within the supplementary S1 file:

Table H: Graph theory cluster coefficient, sparsity level 0.10

| **Measure** | **HC** | **MCI** | **Estimate** | **Statistic** | **P-value*** |
| --- | --- | --- | --- | --- | --- |
| **LH** |  |  |  |  |  |
| DMN-b Temp 3 | 0.558 ± 0.058 | 0.460 ± 0.100 | 0.10 | 4.25 | 0.000114 |
| DMN-a PCC 1 | 0.575 ± 0.079 | 0.490 ± 0.082 | 0.10 | 4.14 | 0.000161 |
| DMN-a PCC 2 | 0.555 ± 0.054 | 0.496 ± 0.075 | 0.07 | 3.70 | 0.000608 |
| SomMot A 16 | 0.531 ± 0.0 | 0.423 ± 0.094 | 0.11 | 3.60 | 0.000808 |
| DMN-b PFCd 1 | 0.530 ± 0.071 | 0.453 ± 0.084 | 0.08 | 3.58 | 0.000871 |
| Hippocampus | 0.498 ± 0.126 | 0.369 ± 0.132 | 0.14 | 3.49 | 0.001137 |
| DMN-a PFCm 3 | 0.561 ± 0.077 | 0.483 ± 0.094 | 0.09 | 3.47 | 0.001198 |
| DMN-b Temp 2 | 0.568 ± 0.089 | 0.478 ± 0.097 | 0.09 | 3.33 | 0.001781 |
| **RH** |  |  |  |  |  |
| Hippocampus | 0.527 ± 0.125 | 0.357 ± 0.136 | 0.20 | 5.53 | 0.000002 |
| DMN-a PFCm 1 | 0.536 ± 0.068 | 0.450 ± 0.080 | 0.09 | 4.12 | 0.000170 |
| DMN-b PFCd 4 | 0.555 ± 0.076 | 0.475 ± 0.107 | 0.10 | 3.72 | 0.000572 |
| Limibic-a OFC 6 | 0.553 ± 0.079 | 0.453 ± 0.112 | 0.11 | 3.60 | 0.000818 |
| SalVentAttn A ParMed 8 | 0.500 ± 0.101 | 0.426 ± 0.102 | 0.10 | 3.49 | 0.001124 |
| SalVentAttn A ParMed 4 | 0.487 ± 0.094 | 0.399 ± 0.090 | 0.10 | 3.46 | 0.001235 |
| DMN-a PFCd 2 | 0.555 ± 0.078 | 0.463 ± 0.095 | 0.09 | 3.45 | 0.001268 |
| DMN-a PCC 1 | 0.559 ± 0.064 | 0.494 ± 0.077 | 0.07 | 3.36 | 0.001649 |

** p*-values are uncorrected 2-sided

Graph theory analysis, at sparsity level 0.10, revealed a significant between-group difference in the cluster coefficient measure. This was higher in the HC (M=0.484, SD=0.027) compared to the MCI (M=0.461, SD=0.035) group (**b** = .03, *t* = 2.90, *p*_uncor_ = .006). See table H above.

Graph theory analysis, at sparsity level 0.20, revealed a significant between-group difference in the cluster coefficient measure. This was higher in the HC (M=0.504, SD=0.025) compared to the MCI (M=0.490, SD=0.031) group (**b** = .02, *t* = 2.31, *p*_uncor_ = .026). The ROIs driving this difference did not survive correction for multiple comparisons.

Graph theory analysis, at sparsity level 0.25, revealed a significant between-group difference in the cluster coefficient measure. This was higher in the HC (M=0.521, SD=0.024) compared to the MCI (M=0.509, SD=0.029) group (**b** = .02, *t* = 2.19, *p*_uncor_ = .034). See table I.

Table I: Graph theory cluster coefficient, sparsity level 0.25

| **Measure** | **HC** | **MCI** | **Estimate** | **Statistic** | **P-value*** |
| --- | --- | --- | --- | --- | --- |
| **LH** |  |  |  |  |  |
| DMN-b Temp 3 | 0.580 ± 0.051 | 0.512 ± 0.070 | 0.08 | 4.69 | 0.00028 |

** p*-values are uncorrected 2-sided

Graph theory analysis, at sparsity level 0.10, revealed a statistical difference in average path length between the groups, with a longer path length in HC compared to controls (*b* = .05, *t* = 2.42, *p*_uncor_ = .020; HC: M = 2.39, SD = 0.071; MCI: M = 2.35, SD = 0.084). This difference was driven by two ROIs in the somatomotor network - one in the left hemisphere and one in the right.

Table J: Graph theory average path length, sparsity level 0.10

| **Measure** | **HC** | **MCI** | **Estimate** | **Statistic** | **P-value*** |
| --- | --- | --- | --- | --- | --- |
| **LH** |  |  |  |  |  |
| SomMot-a 15 | 2.46 ± 0.215 | 2.22. ± 0.180 | 0.25 | 4.25 | 0.000112 |
| **RH** |  |  |  |  |  |
| SomMot-a 13 | 2.45 ± 0.129 | 2.25 ± 0.200 | 0.19 | 4.20 | 0.000133 |

** p*-values are uncorrected 2-sided

Graph theory analysis did not reveal a stastical difference in average path length between the two groups at sparsity level 0.20: (*b* = .02, *t* = 1.54, *p*_uncor_ = .130; HC: M = 1.89, SD = 0.035; MCI: M = 1.88, SD = 0.040), nor at sparsity level 0.25: (*b* = .01, *t* = 1.11, *p*_uncor_ = .275; HC: M = 1.78, SD = 0.024; MCI: M = 1.78, SD = 0.029).

**2. L292: “Within hemisphere only analyses were conducted, across hemisphere connectivity was not inspected due to sample size”. It’s better and possible to make a comparison across hemisphere because authors didn’t use multivariate.**

We agree with the reviewer that such comparisons of functional connectivity are of interest. However, we were confronted with the statistical limitations of our sample size. With seven regions of interest per hemisphere, it would require 91 comparisons to inspect all possible pairings within and across hemispheres. This, alongside the requisite correction for multiple comparisons, was deemed a-priori to be beyond the capacity of this sample’s data.

It is also worth highlighting that analysis of the limbic white matter revealed differences between the hemispheres. However, because the focus was on limbic white matter tracts we did not analyse the corpus callosum. The aforementioned statistical considerations aside, these points taken together, would complicate any interpretation of across hemisphere functional relationships (if we had found them).

**3. L272: “Its lmrob function fits a model based on an M-estimator using iteratively reweighted least squares estimation”. Could you list the model equation in details?**

Here is an indicative example of how the formula was run in R:

lmrob(Mean_FA ~ Group + Age + Gender + Education, data = L_)

This approach was chosen because it is more appropriate for small sample sizes and the attendant problems of meeting test assumptions, including the issue of having a large number of parameters or covariates to model relative to the sample size, as per this study. This approach was chosen based on the recommendation of Field and Wilcox (2017):

“M-estimators determine whether a score is an outlier empirically and if it is, adjustments are made for it. The adjustment could be to completely ignore the observation or to down-weight it. Obvious advantages of M-estimators are that you can (1) down-weight rather than exclude observations; (2) avoid over- or under-trimming your data; and (3) perform non-symmetric trimming.” (p 23, 1)

1. Field AP, Wilcox RR. Robust statistical methods: A primer for clinical psychology and experimental psychopathology researchers. Behav Res Ther [Internet]. 2017;98:19–38. Available from: http://dx.doi.org/10.1016/j.brat.2017.05.013

“This function computes an MM-type regression estimator as described in Yohai (1987) and Koller and Stahel (2011). By default it uses a bi-square redescending score function, and it returns a highly robust and highly efficient estimator (with 50% breakdown point and 95% asymptotic efficiency for normal errors).” (p 67, 2)

2. Maecheler M, Rousseeuw P, Croux C, Todorov V, Rucksuhl A, Salibian-Barrera M, et al. “robustbase”: Basic robust statistics. R package [Internet]. 2018. Available from: http://cran.r-project.org/package=robustbase

A number of formulae are required to calculate the robust regression. With due deference to the field of the statistics, we refer the reviewer to the source materials for a fuller appreciation of the approach:

3. Koller M, Stahel WA. Sharpening Wald-type inference in robust regression for small samples. Comput Stat Data Anal [Internet]. 2011;55(8):2504–15. Available from: http://dx.doi.org/10.1016/j.csda.2011.02.014

4. Yohai, V., 1987. High breakdown-point and high efficiency robust estimates for regression. The Annals of Statistics 15 (2), 642–656.

Minor concerns:
**1. the figures aren’t with enough resolution, especially the title and number are unclear.**

Thank you for highlighting this frustration. We followed the guidelines provided and used the recommended PACE website to ensure the figures met publication standards. Should the paper be accepted, perhaps this is something we can work with the publication team to resolve?

**2. Table 3,4, why t(43), what does 43 mean? And the table format should be revised for consistency.**

t(43) refers to the t-statistic for 43 degrees of freedom (allowing for 2 groups, with age, gender and education as control covariates). As suggested, we have changed the text to ‘statistic’ so that it is consistent with the other tables.

**3. Some expressions are unclear, e.g.**
**L54: “……. more so than local neuropathological processes”.**

We have revised the sentence to improve clarity:

“For example, it is proposed that early hypometabolism seen in the posterior cingulate cortex reflects *distant* damage in the hippocampal formation more so than *local* neuropathological processes within the posterior cingulate cortex (9,10).”

**L91: “ and to a lesser extent in MCI, found using a graph theory approach ”**

We have revised the sentence to improve clarity:

“That finding of increased functional connectivity parallels the lack of segregation between the DMN and frontoparietal networks that have been revealed using a graph theory approach in AD, and to a lesser extent, MCI patients.”

**Reviewer #2:**

**The study used multimodal MRI data including DWI and resting state MRI to compared the difference between MCI and AD. This study has some strengths, such as the advanced analytical method. But there are still several important details need to be clarified.**

Overall Reply: We thank Dr Yue for their comments, and their appreciation and help in producing the improvements made to the manuscript.

For the sake of avoiding confusion in the below responses (in case the paper is not to hand), we wish to clarify that the study compared differences between a group with MCI and a matched healthy control group.

**1. The concept of preclinical stage of AD is definitely not MCI. Cited from a review (Epelbaum et al., 2017), three main issues concerning the concept of preclinical AD must be clarified: 1) the level of cognitive performance considered as normal cognition, 2) the changes in cognitive performance considered as cognitive decline, and 3) the best biomarkers or the best combination of them able to identify the “AD pathophysiological signature” in vivo. Hence, the stage of MCI should be prodromal AD, but not preclinical. Please clarify all related content.
Ref: Epelbaum S, Genthon R, Cavedo E, et al. Preclinical Alzheimer's disease: A systematic review of the cohorts underlying the concept [J]. Alzheimers Dement, 2017, 13(4): 454-67.**

Thank you for pointing this out and for providing the relevant reference. We have removed the two references to preclinical AD, in the title and in the abstract (line 15), and replaced with prodromal AD:

Title: “No relationship between fornix and cingulum degradation and within-network decreases in functional connectivity in prodromal Alzheimer’s disease”

Line 15: “We investigated changes in functional and structural connectivity among regions that support memory function in prodromal Alzheimer’s disease, i.e., during the mild cognitive impairment (MCI) stage.”

**2. In the section of Introduction, I think it would have been better to propose the research aims at the last paragraph after review all the related studies and their limitation.**

We agree that is helpful to place the purpose of the study at the end of the introduction section. We had previously received feedback from colleagues urging us to provide context in the initial paragraph of the introduction. It seems to be an important point for the reader. For these reasons we attempted to address both viewpoints by mentioning aims in both the first and last paragraph of the introduction:

First paragraph of introduction:

“This study aims to probe the relationship between structural and functional brain changes in MCI through use of resting state magnetic resonance imaging (rsMRI) and diffusion weighted imaging (DWI).”

Last paragraph of introduction:

“This study adds to the literature by investigating constrained spherical deconvolution DWI measures (54) of MCI-targeted white matter tracts (fornix, cingulum bundles, and uncinate fasciculus) and temporal correlation connectivity measures of MCI-targeted functional networks (DMN and limbic), and by examining the relationship between those structural and functional measures.”

Admittedly this approach is unlikely to be agreeable to all stylistic perspectives. However, we hope it does not deter from the overall readability and understanding of the paper and its aims.

**3. In line 142, the cut-off score in GDS is 5?**

Thank you for picking this up. We used the *short-form* of the GDS, it has a suggested cut-off of 5. We have inserted ‘short-form’ at the first mention of the use of the GDS in our study, in line 151:

‘……short-form Geriatric Depression Scale (GDS; (59))’

**4. In Line 275-277,” We chose not to include depression as a control covariate given that its presence may reflect dementia pathology rather than a reaction to memory problems.” The relationship between depression and cognitive function is very complicated, part of the depressive elderly would never convert to dementia. This sentence is confusing.** **Please clarify and revise related analysis result.**

Thank you for bringing our attention to this section of text. We have inserted the below text within the analysis section. The text now emphasizes the complication of depression and provides more clarity around the reasons for our analysis choice. Now line 299-304:

The relationship between depression and cognitive function is complicated and some depressive elderly will not convert to dementia (**84**). However, we chose not to include depression as a control covariate given that its presence may reflect dementia pathology (85). That is, controlling for depression would run the risk of removing relevant explanatory variance. Further, in this group it would both reduce sample size as one participant declined to complete the GDS and risk over-fitting the model. In any case, in this cohort depression did not correlate with worsening measures of cognition (see Fig B in S1 file).

Further, we have clarified the details on missing neuropsychological information in the methods section:

“Five MCI people declined to complete the entirety of the neuropsychological testing set (four did not complete the CERAD tests, one did not complete the CERAD or GDS tests) – however, all were successfully scanned.”

**5. The author should clarify the definition of DMN-a, -b and -c, because it seems very confusing for a clinician reading.**

Thank you for sharing this perspective. The atlas we used (Schaefer et al, 2018) features a relatively recent and specific computational-based segmentation of the default mode network based on functional connectivity.

These parcellations were defined using task and resting state fMRI data of 1489 young adults (18-35 years old) and a computational method that made the optimal combination of two different segmentation approaches: 1) abrupt changes in functional connectivity patterns such as might be defined histologically and 2) parcels of similar functional activity identified independently of spatial considerations. This parcellation method is thought to reflect functional brain organization in a neurobiologically meaningful way.

Alongside creating functional anatomical clusters, the parcellations are helpful from a research and statistical perspective (it moves on from examining every single voxel in a MRI dataset). In order to address different research needs, the authors provided parcellations in varying amounts of clusters (400, 600, 800, 1000), i.e., from diffuse to focal levels. In our study we chose the atlas with 400 parcellations. In that instance it revealed a default mode network that could be meaningfully sub-divided into 3 functional clusters, and a limbic network that could be divided into two functional clusters. Within each of these clusters focal sources of the functional activity can be further labelled. In figure 2 of the paper, we provided a view of the focal sources within each cluster of the default mode and limbic networks.

We have amended the text as follows:

Using the 2mm 400 region cortical atlas (80) two limbic (a and b) and three default mode sub-networks (a, b, and c) per hemisphere were identified. These atlas parcellations were computed from functional connectivity patterns. The sources of the functional connectivity signals within each sub-network are detailed in Fig 2. Centroid co-ordinates for the parcels are presented in S1 file table L.

Due to its length, table L has been placed at the end of the present document.

Schaefer A, Kong R, Gordon EM, Laumann TO, Zuo X, Holmes AJ, et al. Local-Global Parcellation of the Human Cerebral Cortex from Intrinsic Functional Connectivity MRI. Cereb Cortex. 2018;28:3095–114.

6. **Due to the small sample size, I think it is better to compare with age, gender matched controls. In addition, the age distribution was “54-80”. For neuroimage study, especially AD-related study, age is a very important factor.**

We agree with the reviewer that age is an important consideration. While the healthy control group is unfortunately not a precise like-for-like of the MCI group, there were no statistically significant demographic differences between the two groups. We also took the step of including age, gender and education as control covariates in the linear regressions.

Taking the opportunity to reflect on the reviewer’s comment regarding the sample more generally, we have included an additional sentence (L504) to highlight the possibility that the lack of finding of a relationship between functional and structural connectivity is due to the sample size constraints:

“It is also possible that, notwithstanding the simple statistical approach we used, the sample size is simply too small (see limitations section).”

**7. About method, there are some significant difference between group in neuropsychological test, such as MMSE, word delay test, so what’s the relationship between the neuroimage biomarker with these clinical data. Please do some further correlation analysis which may reflect the clinical implication.**

Thank you for raising this point, we do appreciate that such an analysis could hold interest. We chose not to explore this aspect primarily because it falls outside the focus of the paper. It also risks ending up reporting spurious relations due to statistical limitations. There are a couple of reasons for this. First, there is the potential to investigate multiple relationships – there are 50 metrics for the DWI data and 52 for the functional connectivity data that could each be investigated for relationships with up to 11 neuropsychological measures. Even limiting the analysis to a post-hoc exploration between those imaging and neuropsychological measures that were significantly different between groups would still require 120 tests. Second, as mentioned above, five MCI patients have incomplete neuropsychological test sets thus reducing the sample size available for such correlations to twenty patients. For these reasons, albeit that we appreciate the rationale for the suggestion, we would prefer not to undertake those analyses. The data is being made publicly available and we sincerely hope that it might be of use to other researchers who may be in a position to combine it with other datasets to create the statistical bandwidth to answer such questions.

Reviewer #2, point 5: Additional information for the supplementary file:

Table L: Schaefer atlas 2018 400 parcels 17 networks MNI152 2mm – centroid co-ordinates for default mode and limbic networks. Elements shaded in grey were implicated in one/more graph theory analyses. For complete list of centroids see: https://github.com/ThomasYeoLab/CBIG/blob/master/stable_projects/brain_parcellation/Schaefer2018_LocalGlobal/Parcellations/MNI/Centroid_coordinates/Schaefer2018_400Parcels_17Networks_order_FSLMNI152_2mm.Centroid_RAS.csv

| **ROI Index** | **Label Name** | **X** | **Y** | **Z** |
| --- | --- | --- | --- | --- |
| 36 | 17Networks_LH_SomMotA_12 | -24 | -10 | 64 |
| 39 | 17Networks_LH_SomMotA_15 | -4 | -26 | 68 |
| 40 | 17Networks_LH_SomMotA_16 | -14 | -12 | 72 |
| 109 | 17Networks_LH_Limbic_OFC_1 | -12 | 24 | -20 |
| 110 | 17Networks_LH_Limbic_OFC_2 | -24 | 22 | -20 |
| 111 | 17Networks_LH_Limbic_OFC_3 | -10 | 48 | -22 |
| 112 | 17Networks_LH_Limbic_OFC_4 | -4 | 24 | -20 |
| 113 | 17Networks_LH_Limbic_OFC_5 | -16 | 64 | -8 |
| 114 | 17Networks_LH_Limbic_TempPole_1 | -38 | -6 | -42 |
| 115 | 17Networks_LH_Limbic_TempPole_2 | -24 | 6 | -40 |
| 116 | 17Networks_LH_Limbic_TempPole_3 | -26 | -10 | -32 |
| 117 | 17Networks_LH_Limbic_TempPole_4 | -54 | -22 | -30 |
| 118 | 17Networks_LH_Limbic_TempPole_5 | -40 | -22 | -26 |
| 119 | 17Networks_LH_Limbic_TempPole_6 | -32 | 12 | -30 |
| 120 | 17Networks_LH_Limbic_TempPole_7 | -44 | 6 | -16 |
| 149 | 17Networks_LH_DefaultA_IPL_1 | -48 | -64 | 32 |
| 150 | 17Networks_LH_DefaultA_IPL_2 | -42 | -72 | 44 |
| 151 | 17Networks_LH_DefaultA_PFCd_1 | -24 | 28 | 44 |
| 152 | 17Networks_LH_DefaultA_PFCd_2 | -18 | 36 | 48 |
| 153 | 17Networks_LH_DefaultA_PFCd_3 | -22 | 20 | 52 |
| 154 | 17Networks_LH_DefaultA_PCC_1 | -4 | -54 | 20 |
| 155 | 17Networks_LH_DefaultA_PCC_2 | -6 | -60 | 30 |
| 156 | 17Networks_LH_DefaultA_PCC_3 | -8 | -44 | 32 |
| 157 | 17Networks_LH_DefaultA_PCC_4 | -4 | -34 | 38 |
| 158 | 17Networks_LH_DefaultA_PCC_5 | -2 | -16 | 38 |
| 159 | 17Networks_LH_DefaultA_PCC_6 | -2 | -68 | 42 |
| 160 | 17Networks_LH_DefaultA_PCC_7 | -6 | -50 | 42 |
| 161 | 17Networks_LH_DefaultA_PFCm_1 | -4 | 56 | -10 |
| 162 | 17Networks_LH_DefaultA_PFCm_2 | -6 | 36 | -8 |
| 163 | 17Networks_LH_DefaultA_PFCm_3 | -6 | 60 | 6 |
| 164 | 17Networks_LH_DefaultA_PFCm_4 | -6 | 44 | 6 |
| 165 | 17Networks_LH_DefaultA_PFCm_5 | -16 | 68 | 8 |
| 166 | 17Networks_LH_DefaultA_PFCm_6 | -6 | 34 | 20 |
| 167 | 17Networks_LH_DefaultB_Temp_1 | -44 | 12 | -34 |
| 168 | 17Networks_LH_DefaultB_Temp_2 | -54 | -2 | -30 |
| 169 | 17Networks_LH_DefaultB_Temp_3 | -62 | -18 | -20 |
| 170 | 17Networks_LH_DefaultB_Temp_4 | -56 | -8 | -14 |
| 171 | 17Networks_LH_DefaultB_Temp_5 | -60 | -34 | -4 |
| 172 | 17Networks_LH_DefaultB_Temp_6 | -52 | -22 | -6 |
| 173 | 17Networks_LH_DefaultB_IPL_1 | -46 | -58 | 20 |
| 174 | 17Networks_LH_DefaultB_IPL_2 | -56 | -54 | 30 |
| 175 | 17Networks_LH_DefaultB_PFCd_1 | -4 | 52 | 28 |
| 176 | 17Networks_LH_DefaultB_PFCd_2 | -14 | 58 | 30 |
| 177 | 17Networks_LH_DefaultB_PFCd_3 | -22 | 50 | 32 |
| 178 | 17Networks_LH_DefaultB_PFCd_4 | -8 | 42 | 52 |
| 179 | 17Networks_LH_DefaultB_PFCd_5 | -12 | 24 | 60 |
| 180 | 17Networks_LH_DefaultB_PFCd_6 | -6 | 10 | 64 |
| 181 | 17Networks_LH_DefaultB_PFCl_1 | -40 | 20 | 48 |
| 182 | 17Networks_LH_DefaultB_PFCl_2 | -42 | 8 | 48 |
| 183 | 17Networks_LH_DefaultB_PFCv_1 | -36 | 22 | -16 |
| 184 | 17Networks_LH_DefaultB_PFCv_2 | -36 | 36 | -12 |
| 185 | 17Networks_LH_DefaultB_PFCv_3 | -46 | 32 | -10 |
| 186 | 17Networks_LH_DefaultB_PFCv_4 | -48 | 28 | 0 |
| 187 | 17Networks_LH_DefaultB_PFCv_5 | -54 | 20 | 12 |
| 188 | 17Networks_LH_DefaultC_IPL_1 | -40 | -78 | 30 |
| 189 | 17Networks_LH_DefaultC_Rsp_1 | -14 | -48 | 4 |
| 190 | 17Networks_LH_DefaultC_Rsp_2 | -8 | -52 | 10 |
| 191 | 17Networks_LH_DefaultC_Rsp_3 | -14 | -60 | 18 |
| 192 | 17Networks_LH_DefaultC_PHC_1 | -20 | -20 | -26 |
| 193 | 17Networks_LH_DefaultC_PHC_2 | -30 | -32 | -18 |
| 194 | 17Networks_LH_DefaultC_PHC_3 | -18 | -38 | -12 |
| 236 | 17Networks_RH_SomMotA_13 | 10 | -40 | 68 |
| 299 | 17Networks_RH_SalVentAttnA_ParMed_4 | 6 | 10 | 58 |
| 302 | 17Networks_RH_SalVentAttnA_ParMed_7 | 6 | -2 | 66 |
| 303 | 17Networks_RH_SalVentAttnA_ParMed_8 | 16 | 6 | 70 |
| 313 | 17Networks_RH_Limbic_OFC_1 | 14 | 24 | -20 |
| 314 | 17Networks_RH_Limbic_OFC_2 | 22 | 22 | -20 |
| 315 | 17Networks_RH_Limbic_OFC_3 | 8 | 46 | -24 |
| 316 | 17Networks_RH_Limbic_OFC_4 | 20 | 42 | -18 |
| 317 | 17Networks_RH_Limbic_OFC_5 | 4 | 22 | -20 |
| 318 | 17Networks_RH_Limbic_OFC_6 | 10 | 62 | -14 |
| 319 | 17Networks_RH_Limbic_TempPole_1 | 28 | -2 | -40 |
| 320 | 17Networks_RH_Limbic_TempPole_2 | 48 | -6 | -40 |
| 321 | 17Networks_RH_Limbic_TempPole_3 | 36 | 18 | -38 |
| 322 | 17Networks_RH_Limbic_TempPole_4 | 40 | -14 | -32 |
| 323 | 17Networks_RH_Limbic_TempPole_5 | 28 | 12 | -30 |
| 324 | 17Networks_RH_Limbic_TempPole_6 | 50 | -28 | -26 |
| 358 | 17Networks_RH_DefaultA_Temp_1 | 60 | -8 | -24 |
| 359 | 17Networks_RH_DefaultA_IPL_1 | 54 | -54 | 26 |
| 360 | 17Networks_RH_DefaultA_IPL_2 | 48 | -64 | 42 |
| 361 | 17Networks_RH_DefaultA_PFCd_1 | 26 | 34 | 38 |
| 362 | 17Networks_RH_DefaultA_PFCd_2 | 24 | 26 | 50 |
| 363 | 17Networks_RH_DefaultA_PCC_1 | 6 | -52 | 24 |
| 364 | 17Networks_RH_DefaultA_PCC_2 | 4 | -64 | 32 |
| 365 | 17Networks_RH_DefaultA_PCC_3 | 6 | -38 | 34 |
| 366 | 17Networks_RH_DefaultA_PCC_4 | 4 | -20 | 36 |
| 367 | 17Networks_RH_DefaultA_PCC_5 | 10 | -52 | 36 |
| 368 | 17Networks_RH_DefaultA_PFCm_1 | 6 | 42 | -10 |
| 369 | 17Networks_RH_DefaultA_PFCm_2 | 10 | 66 | 0 |
| 370 | 17Networks_RH_DefaultA_PFCm_3 | 8 | 42 | 4 |
| 371 | 17Networks_RH_DefaultA_PFCm_4 | 8 | 54 | 12 |
| 372 | 17Networks_RH_DefaultA_PFCm_5 | 18 | 64 | 16 |
| 373 | 17Networks_RH_DefaultA_PFCm_6 | 6 | 26 | 18 |
| 374 | 17Networks_RH_DefaultB_Temp_1 | 64 | -24 | -8 |
| 375 | 17Networks_RH_DefaultB_Temp_2 | 64 | -38 | 0 |
| 376 | 17Networks_RH_DefaultB_AntTemp_1 | 50 | 8 | -32 |
| 377 | 17Networks_RH_DefaultB_PFCd_1 | 6 | 58 | 30 |
| 378 | 17Networks_RH_DefaultB_PFCd_2 | 16 | 52 | 36 |
| 379 | 17Networks_RH_DefaultB_PFCd_3 | 4 | 44 | 40 |
| 380 | 17Networks_RH_DefaultB_PFCd_4 | 14 | 38 | 52 |
| 381 | 17Networks_RH_DefaultB_PFCd_5 | 12 | 20 | 62 |
| 382 | 17Networks_RH_DefaultB_PFCv_1 | 34 | 22 | -18 |
| 383 | 17Networks_RH_DefaultB_PFCv_2 | 48 | 32 | -8 |
| 384 | 17Networks_RH_DefaultB_PFCv_3 | 54 | 24 | 6 |
| 385 | 17Networks_RH_DefaultC_IPL_1 | 48 | -64 | 22 |
| 386 | 17Networks_RH_DefaultC_IPL_2 | 46 | -76 | 30 |
| 387 | 17Networks_RH_DefaultC_Rsp_1 | 14 | -46 | 4 |
| 388 | 17Networks_RH_DefaultC_Rsp_2 | 12 | -56 | 16 |
| 389 | 17Networks_RH_DefaultC_PHC_1 | 22 | -18 | -28 |
| 390 | 17Networks_RH_DefaultC_PHC_2 | 30 | -30 | -18 |
