# Supplementary material for: No relationship between fornix and cingulum degradation and within-network decreases in functional connectivity in prodromal Alzheimer’s disease
Source: PLoS One. 2019 Oct 3;14(10):e0222977. doi: 10.1371/journal.pone.0222977 (PMC6776361; doi:10.1371/journal.pone.0222977)
Supplement: Attachment — Submitted filename: Response to Reviewers.docx [file pone.0222977.s003.docx]

**We thank both reviewers for again spending time and giving great attention to improving the paper. We are appreciative of your support.**

**Reviewer #1:** Minor concerns:

The sign of static value in different tables may not be consistent. For example, in table 2, positive statistic value means increase in MCI subjects, negative means decrease. But in table 3, they show opposite meaning according to the results description.

The statistic value means T value? It's better to be clearer in each table.

Reply: You are correct. Differences in how the reference group was defined in the various analyses files are behind these inconsistencies. However, as you point out the explanation for said differences had been correctly described in the manuscript.

We have amended the text for the DWI results and its corresponding Table 2 in the main manuscript:

All diffusion measures of the fornix showed evidence of degeneration in the MCI group: MD, Da and Dr were comparatively increased in the left (MD: *t* = 4.21, *p_cor_* = .0006, Da: *t* = 5.32, *p_cor_* = .00005, Dr: *t* = 3.74, *p_cor_* = .002) and the right fornix (MD: *t* = 5.06, *p_cor_* < .0001, Da: *t* = 5.59, *p_cor_* = .00005, Dr: *t* = 4.65, *p_cor_* = .0002), and FA was comparatively decreased in the left (*t* = -2.78, *p_cor_* = .019) and right (*t* = -2.42, *p_cor_* = .035) fornices. In the MCI compared to the HC group, MD and Da were significantly increased in the left parahippocampal cingulum (MD: *t* = 2.72, *p_cor_* = .019, Da: *t* = 3.02, *p_cor_* = .012), and MD and Dr were significantly increased in the left retrosplenial cingulum (MD: t = 3.08, *p_cor_* = .011, Dr: *t* = 2.43, *p_cor_* = .035) and in the left subgenual cingulum (MD: *t* = 3.08, *p_cor_* = .011, Dr: *t* = 2.77, *p_cor_* = .019).

**Table 3. MD values per structure per hemisphere**

| **Structure** | **Hemi** | **HC** | **MCI** | **Estimate** | ***t*-statistic** | **p-value *** |
| --- | --- | --- | --- | --- | --- | --- |
| Fornix | LH | 0.00120 ± 0.00008 | 0.00132 ± 0.00014 | 0.000139 | 4.210 | 0.000064 |
|  | RH | 0.00118 ± 0.00008 | 0.00127 ± 0.00008 | 0.000100 | 5.056 | .0000042 |
| Para-hippocampal cingulum | LH | 0.00072 ± 0.00003 | 0.00075 ± 0.00005 | 0.0000187 | 2.721 | 0.00467 |
|  | RH | 0.00072 ± 0.00003 | 0.00074 ± 0.00005 | 0.0000104 | 1.007 | 0.15950 |
| Retrosplenial cingulum | LH | 0.00067 ± 0.00002 | 0.00069 ± 0.00002 | 0.0000213 | 3.084 | 0.00178 |
|  | RH | 0.00067 ± 0.00002 | 0.00068 ± 0.00003 | 0.0000148 | 2.052 | 0.02315 |
| Subgenual cingulum | LH | 0.00069 ± 0.00002 | 0.00070 ± 0.00002 | 0.00001516 | 3.078 | 0.00181 |
|  | RH | 0.00069 ± 0.00002 | 0.00069 ± 0.00002 | 0.00000946 | 1.706 | 0.04765 |
| Uncinate fasciculus | LH | 0.00070 ± 0.00002 | 0.00071 ± 0.00004 | 0.00000444 | 0.806 | 0.21225 |
|  | RH | 0.00072 ± 0.00002 | 0.00072 ± 0.00003 | 0.00000254 | 0.336 | 0.36900 |

Hemi = hemisphere, LH = left hemisphere, RH = right hemisphere. **p*-values are uncorrected, 1-sided

We have corrected the text for the rsMRI results (tables were already correct):

In MCI compared to HC, statistically smaller within-network connectivity was found in the DMN-a in the left (*t* = -3.38, *p_cor_* = .010) and right (*t* = -3.75, *p_cor_* = .005) hemispheres. See Table 4.

In MCI compared to HC, statistically smaller between-network connectivity was found between DMN-a and DMN-c, between DMN-a and the hippocampus, and between DMN-c and the hippocampus in both the left hemisphere (respectively: *t* = -4.63, *p_cor_* = .002; *t* = -4.21, *p_cor_* = .003; *t* = -3.99, *p_cor_* = .003) and the right hemisphere (respectively: *t* = -3.81, *p_cor_* = .005; *t* = -3.31, *p_cor_* = .011; *t* = -3.52, *p_cor_* = .008). In the right hemisphere only in MCI compared to HC, statistically smaller between-network connectivity was found between DMN-c and the thalamus (*t* = -3.02, *p_cor_* = .021) and between DMN-c and the limbic-a network (*t* = -4.01, *p_cor_* = .003). See Table 5, and Figs 5 and 6, for results mentioned here and Table G in S1 file for the complete set.

We have amended text and table for the graph theory results:

Graph theory analyses, at sparsity level of 0.15, revealed a significant between-group difference in the cluster coefficient measure. This was higher in the HC (M=0.492, SD=0.027) compared to the MCI (M=0.474, SD=0.033) group (**b** = .02, *t* = -2.53, *p*_uncor_ = .015). This difference was driven by 18 ROIs - 8 in DMN-a (5 RH, 3 LH), 5 in DMN-b (2 RH, 3LH), both hippocampi, two ROIs in the LH somatomotor network and one in the RH salient ventral attention network – all of which survived FDR-correction. See Fig 7 and Table 6 for details. The difference in average path length between the two groups did not reach statistical significance (*b* = .03, *t* = -1.94, *p*_uncor_ = .059; HC: M=2.08, SD=0.049; MCI: M=2.06, SD=0.06).

**Table 6. Graph theory cluster coefficient, sparsity level of 0.15**

| **Measure** | **HC** | **MCI** | **Estimate** | ***t*-statistic** | **P-value*** |
| --- | --- | --- | --- | --- | --- |
| **LH** |  |  |  |  |  |
| DMN-a PCC 2 | 0.569 ± 0.045 | 0.510 ± 0.067 | 0.07 | -4.17 | 0.000146 |
| DMN-b Temp 3 | 0.564 ± 0.054 | 0.481 ± 0.096 | 0.09 | -3.98 | 0.000262 |
| DMN-b PFCd 5 | 0.540 ± 0.087 | 0.454 ± 0.089 | 0.10 | -3.79 | 0.000458 |
| DMN-a PFCm 1 | 0.536 ± 0.065 | 0.471 ± 0.066 | 0.07 | -3.65 | 0.000713 |
| DMN-a PFCd 3 | 0.525 ± 0.073 | 0.469 ± 0.071 | 0.07 | -3.53 | 0.001002 |
| DMN-b PFCd 1 | 0.543 ± 0.065 | 0.471 ± 0.075 | 0.08 | -3.49 | 0.001117 |
| SomMoTA 16 | 0.546 ± 0.089 | 0.458 ± 0.104 | 0.10 | -3.42 | 0.001383 |
| Hippocampus | 0.501 ± 0.094 | 0.429 ± 0.081 | 0.09 | -3.35 | 0.001687 |
| SomMoTA 12 | 0.544 ± 0.089 | 0.466 ± 0.089 | 0.09 | -3.29 | 0.002004 |
| **RH** |  |  |  |  |  |
| DMN-a PFCm 1 | 0.532 ± 0.062 | 0.451 ± 0.071 | 0.09 | -4.77 | 0.000022 |
| DMN-a PFCd 2 | 0.559 ± 0.061 | 0.466 ± 0.080 | 0.10 | -4.46 | 0.000058 |
| Hippocampus | 0.514 ± 0.123 | 0.421 ± 0.093 | 0.11 | -3.78 | 0.000472 |
| DMN-a PFCm 4 | 0.542 ± 0.062 | 0.477 ± 0.071 | 0.07 | -3.52 | 0.001023 |
| DMN-b Ant Temp 1 | 0.541 ± 0.063 | 0.467 ± 0.096 | 0.09 | -3.51 | 0.001052 |
| DMN-b PFCd 4 | 0.558 ± 0.066 | 0.488 ± 0.101 | 0.09 | -3.46 | 0.001215 |
| DMN-a PCC 1 | 0.573 ± 0.057 | 0.512 ± 0.069 | 0.07 | -3.46 | 0.001222 |
| DMN-a PFCm 3 | 0.507 ± 0.086 | 0.452 ± 0.075 | 0.08 | -3.43 | 0.001335 |
| SalVentAttn A ParMed7 | 0.497 ± 0.067 | 0.437 ± 0.057 | 0.06 | -3.34 | 0.001735 |

** p*-values are uncorrected 2-sided

Any inconsistencies in the S1 file have also been rectified.

As recommended, we have replaced the column header ‘statistic’ with ‘t-statistic’ in the relevant tables throughout the main manuscript and the S1 file.

Reviewer #2:

The authors addressed all the comments adequately, and their manuscript is significantly improved. I have no further comments.
